# Exploring Beverage Intake and Dietary Timing Patterns in Medication-Induced Xerostomia: A Cross-Sectional Pilot Study

**DOI:** 10.3390/nu18040661

**Published:** 2026-02-18

**Authors:** Alhanoof K. Alarfaj, Abdul B. Barmak, Waldir M. Filho, Szilvia Arany

**Affiliations:** 1 Department of General Dentistry, Eastman Institute for Oral Health, University of Rochester, Rochester, NY 14620, USA; 2 Eastman Institute for Oral Health, University of Rochester, Rochester, NY 14620, USA; 3 Specialty Care, Department of Dentistry, Eastman Institute for Oral Health, University of Rochester, 625 Elmwood Avenue, Rochester, NY 14620, USA

**Keywords:** xerostomia, beverage, intake, meal timing, saliva flow, medication

## Abstract

**Background:** Xerostomia, or the subjective sensation of dry mouth, is a prevalent side effect of medications. While its impact on oral function is well documented, limited research has examined how xerostomia influences beverage consumption and the timing of food intake, especially in non-cancer, middle-aged populations. **Objectives:** To examine associations between beverage intake, meal timing, and subjective and objective measures of salivary gland function in individuals with medication-induced xerostomia. **Methods:** This cross-sectional pilot study included 90 adults aged 45–66 taking anticholinergic medications. Salivary function was assessed via unstimulated whole saliva (UWS), minor salivary flow (MSF), and the Xerostomia Inventory (XI). Validated questionnaires evaluated habitual water and beverage intake, and meal timing. Multivariable models were adjusted for demographics, medication use, and comorbidities. **Results:** Hyposalivation (UWS ≤ 0.3 mL/min) was associated with higher XI scores (*p* = 0.033) and greater anticholinergic medication burden (*p* = 0.024). The later timing of last intake and last snack was independently associated with greater xerostomia severity. Total beverage and water intake were not associated with salivary flow or XI scores. Nighttime eating was correlated with higher anticholinergic burden. **Conclusions:** The timing of intake, rather than fluid volume, may better reflect symptom burden in medication-induced xerostomia, underscoring the behavioral adaptations to oral dryness.

## 1. Introduction

Despite growing interest in the complex reciprocal relationship between dietary intake, and systemic and oral health [1,2], little is known about habitual beverage intake, including water, among patients with dry mouth. The subjective perception of dry mouth, aka xerostomia, is one of the most prevalent oral health issues, with incidence increasing with age [3,4], affecting nearly one in three individuals [5,6], and is primarily associated with medications that interfere with cholinergic stimulation of salivary gland secretion [7,8,9]. The feeling of dry mouth often corresponds to, but may not coincide with, hyposalivation, the objective reduction in the salivary flow rate [10,11]. Dry mouth has been shown to impair mastication and tasting, thereby altering eating behavior [12,13,14]. The insufficient lubrication effect of saliva has been shown to compromise intraoral mucosal protection and to hamper oral processing of food, the initial stages of enzymatic digestion, bolus cohesion, and flavor perception [15,16,17]. Associations between dry mouth and malnutrition or reduced dietary intake have been reported in older adults [18,19]. Individuals with dry mouth often modify their diets by avoiding dry or abrasive foods and adjusting their beverage intake during and between meals. Additionally, dry mouth has been found to lead to compensatory dietary and drinking patterns, particularly in the evenings and at night [20]. Only a few studies have examined dietary patterns in individuals with medication-induced xerostomia [21]. The role of beverage and water intake has received little attention, and to date, only a few investigations have integrated either validated dietary assessment tools or utilized objective measures of salivary gland secretion (sialometry) [22,23].

Previous studies investigating the relationship between dietary choices and dry mouth have mainly relied on subjective assessments of dry mouth or focused on oncologic populations, primarily head and neck cancer [24]. Evidence from individuals with medication-induced xerostomia remains limited in non-geriatric populations and in patients without a history of cancer or exposure to cancer therapies, where dry mouth can be attributed specifically to pharmacological causes. To address these gaps, this exploratory study examined habitual beverage consumption and the timing of food intake among individuals with medication-induced xerostomia. Participants’ dry mouth status was assessed using comprehensive subjective and objective measures of salivary gland function. Specifically, salivary flow rates were measured for whole unstimulated saliva and minor salivary glands, and xerostomia severity was evaluated using a validated, widely accepted 11-item questionnaire. Additionally, we explored potential disruptions in meal timing and assessed whether individuals with confirmed hyposalivation engage in irregular meal timing, such as late-night eating and prolonged mealtime windows. Our study examined whether meal timing or beverage intake was independently associated with saliva secretion rates and xerostomia severity, after adjusting for age, biological sex, medication use, BMI, and comorbidities that may potentially affect dietary intake (diabetes, thyroid disease, gastrointestinal conditions). We hypothesized that habitual beverage intake and meal timing patterns would differ according to subjective and objective dry mouth status.

## 2. Materials and Methods

### 2.1. Study Design and Population

This cross-sectional, explorative investigation was conducted as part of an ongoing prospective clinical study examining the impact of anticholinergic medications on salivary gland function. Adult individuals aged 45–66 years were participants of that IRB-approved investigation (#5666) conducted at the Eastman Institute for Oral Health. The study adhered to the principles outlined in the Declaration of Helsinki. The middle-aged range was selected to minimize confounding from age-related changes while capturing a study sample with a high prevalence of medication use [25]. To reduce bias and isolate the effect of medication-induced xerostomia, our study excluded individuals with Sjögren’s syndrome, cancer and radiation therapy, salivary gland surgery, systemic disorders affecting salivary gland function, active use of cholinergic agonists, or systemic conditions known to directly impair salivary gland function. Eligible participants were required to have a verified medication list and to be taking at least one anticholinergic medication for at least 30 days prior to assessment. Eligible participants who expressed interest were enrolled after providing informed consent electronically. Informed consent to participate was obtained from all the participants in the study. During the study visit, participants completed study questionnaires, and demographic variables, including age and biological sex, were self-reported. Additional clinical data, such as BMI and complete medical history, including medications and anticholinergic burden, were cross-verified with electronic health records to ensure accuracy and completeness. The anticholinergic burden from medications was estimated using a modified, dose-weighted version of the Anticholinergic Drug Scale (ADS) [26].

### 2.2. Assessment of Xerostomia

Xerostomia was assessed using the Xerostomia Inventory (XI) [27], a validated 11-item questionnaire designed to assess the severity and frequency of dry mouth symptoms. The total XI score ranges from 11 to 55, with higher scores reflecting a greater severity of xerostomia.

### 2.3. Sialometry

A standardized sialometry protocol included measuring unstimulated whole saliva (UWS) and minor salivary gland flow (MSF) rates between 9:00 a.m. and 12:00 p.m. to minimize circadian variation in salivary secretion. Participants were instructed to refrain from eating, drinking, smoking, brushing their teeth, using mouthwash, chewing gum, or rinsing their mouths for at least one hour prior to the visit. UWS was collected under resting conditions [28]. Participants were instructed to accumulate saliva in their mouths and collect it into pre-weighed, sterile plastic tubes over a 10 min period while sitting upright. The collected samples were weighed using a calibrated analytical balance, and salivary volume was calculated based on a specific gravity of 1.0. Flow rates were expressed in milliliters per minute (mL/min). For assessing salivary flow status, hyposalivation was defined as a UWS flow rate ≤0.3 mL/min, based on established reference values reported in prior studies [4,29]. Flow rates ≤0.1 mL/min were considered severe hyposalivation [21]. During the MSF assessment of the upper lip, secretion from the parotid ducts was blocked bilaterally using cotton rolls [30]. A filter paper strip (5 × 35 mm) was placed horizontally under the upper lip, without contacting the teeth or gingiva. Participants were instructed to avoid lip or mouth movements during the measurement period to prevent mechanical stimulation of saliva secretion. The strip was pre-weighed and immediately re-weighed after collection (1 min) using an analytical microbalance to determine the volume of absorbed saliva. The flow rate was calculated by subtracting the paper weight before collection from the weight after collection, then normalized to the total area and the collection time, expressed in microliters per square centimeter per minute (µL/cm^2^/min).

### 2.4. Dietary Assessment

Dietary habits were evaluated using validated questionnaires in the oral health context, including a beverage intake questionnaire to assess total beverage and water consumption and a food timing screener to evaluate meal frequency and timing. Beverage consumption was assessed using the Beverage Intake Questionnaire (BEVQ-15), a validated 15-item, self-administered screener that estimates habitual intake of commonly consumed beverage categories over the past month [31]. The instrument captures both frequency and volume for each beverage type, enabling estimation of total fluid volume and beverage-derived caloric intake. For this analysis, three continuous variables were derived: total beverage volume (mL/day), total plain water intake (mL/day), and total beverage-derived energy intake (kcal/day). These were selected based on their hypothesized relevance to salivary function and xerostomia. Computation of each variable followed established scoring procedures, with water defined as plain drinking water, beverage volume as the sum of all beverage categories, and beverage kcal derived from caloric beverages (e.g., sugar-sweetened, and alcoholic drinks). 

Meal timing variables were assessed using the validated Food Timing Screener (FTS), a structured, self-administered questionnaire designed to capture the habitual timing of food intake and sleep across workdays and non-workdays [32]. The FTS instrument has previously demonstrated adequate reliability and utility for identifying lifestyle contributors to circadian disruption in clinical populations and includes time entries for meals and snacks, as well as usual wake and sleep times, allowing for the calculation of derived intake-timing variables. Consistent with the original validation and prior applied studies [33], five variables were generated for analysis: first intake time, last intake time, total eating/drinking window, intake after 9 p.m. (yes/no), and nighttime intake (yes/no). Eating window classifications were defined based on the prior literature. A short eating window was defined as less than 12 h, reflecting durations associated with beneficial behavioral and metabolic outcomes in previous studies [34]. Conversely, a long eating window was defined as greater than 14 h [35]. Irregular food timing was defined as a difference of more than two hours in the first or last intake between workdays and free days.

### 2.5. Sample Size and Power Considerations

This study included a total of 90 participants who provided responses to the dietary intake questionnaire, reflecting the full available sample at the time of this pilot data collection. The sample size was not based on an a priori power calculation; however, a post hoc power analysis was conducted to assess the study’s sensitivity to detect meaningful effects. Given α = 0.05 (two-tailed), the sample size of 90 provided 80% power to detect Spearman correlation coefficients of approximately ρ = 0.29 or greater, and group differences corresponding to Cohen’s d ≥ 0.65, accounting for the unequal group sizes. While the study may have been underpowered to detect small effects, the sample was deemed sufficient for the exploratory and hypothesis-generating objectives.

### 2.6. Statistical Analysis

The normality of continuous variables was assessed using the Shapiro–Wilk test and visual inspection of histograms. Participants were classified into hyposalivation and normal salivation groups, based on UWS flow rate, using a cutoff of ≤0.3 mL/min to define hyposalivation. Mann–Whitney U tests were used to compare continuous variables between groups, and Fisher’s exact test was used for categorical variables. All continuous data are reported as median (interquartile range) and 95% confidence intervals. Univariate analyses were conducted to assess associations between dietary intake variables and xerostomia severity (XI), unstimulated whole salivary flow (UWS), and minor salivary gland flow. Depending on variable type, we used Spearman’s rank correlation for continuous predictors, the Mann–Whitney U test for binary categorical variables, and the Kruskal–Wallis test for categorical variables with more than two groups. Multivariable regression analyses were conducted to examine associations between dietary intake behaviors and salivary outcomes. Multivariable linear regression models were used for continuous salivary outcomes, including XI, UWS, and MSF. Each dietary intake variable (e.g., timing of first intake, timing of last intake, snack count, intake window characteristics, water intake, total beverage volume, and total beverage energy intake) was modeled separately in its own regression model for each salivary outcome. Binomial logistic regression models were used to examine associations between dietary intake variables and UWS status (hyposalivation vs. normosalivation). All multivariable models were adjusted for age, biological sex, BMI, ADS, total number of medications, diabetes, endocrine or thyroid disorders, and gastrointestinal conditions. Model assumptions were evaluated using appropriate diagnostic procedures, including assessment of normality, collinearity, and model fit. Collinearity was assessed using variance inflation factors (VIFs), and logistic regression model performance was evaluated using classification accuracy and receiver operating characteristic (ROC) curve analyses. All statistical analyses were conducted using a graphical statistical software interface built on R (4.5.2). Statistical significance was set at *p* < 0.05.

## 3. Results

Table 1 presents clinical and demographic characteristics stratified by salivary flow status.

Compared to participants with normal salivation, those with hyposalivation had a significantly higher number of total medications (*p* = 0.0240), greater prevalence of hormonal disease (e.g., thyroid disease, *p* = 0.0323), lower UWS flow rate (*p* < 0.001), lower MSF rate (*p* < 0.001), and higher XI scores (*p* = 0.0326). No significant group differences were observed in age, BMI, anticholinergic burden, or the prevalence of other medical conditions.

Table 2 presents a comparison of dietary timing and beverage intake characteristics by salivary flow status. We found no significant differences in meal timing variables between workdays and non-workdays. Most participants (94.4%) exhibited consistent food timing throughout workdays and free days, while only a small subset (5.6%) was classified as having irregular food timing, defined as a shift of greater than 2 h between these day types. No statistically significant differences were observed between hyposalivation and normosalivation across any of the measured variables. This includes total water and beverage intake, energy from beverages, the timing of first and last intake, snack frequency, nighttime intake behaviors, and intake window duration and pattern. Breakfast consumption was reported by 66.7% of participants with hyposalivation and 72.5% of those with normal salivation.

Univariate analyses were conducted to examine whether nighttime intake, dietary timing, and beverage intake were associated with salivary function parameters. Participants reporting nighttime intake had more severe dryness (higher XI) and had a higher MSF rate, although these findings are not statistically significant. Participants reporting nighttime intake (13.3% of the sample) exhibited a significantly higher anticholinergic burden (ADS) compared to those without nighttime intake (median ADS: 4 vs. 3; *p* = 0.037). Spearman’s rank-order correlation analyses were conducted to assess the associations between salivary outcomes and various dietary timing and intake variables. There were no statistically significant associations between any salivary outcomes (UWS, MSF, XI) and the dietary timing or intake variables. However, several correlations involving xerostomia severity and intake timing or volume approached significance, suggesting potential underlying patterns. To further explore whether these associations were more pronounced in individuals with clinically defined severe hyposalivation (UWS ≤ 0.1 mL/min, *n* = 29), a subgroup analysis was conducted. In cases of severe hyposalivation, UWS flow was positively correlated with the later timing of last intake (ρ = 0.42, *p* = 0.031), and XI showed trends toward associations with total beverage volume (ρ = 0.37, *p* = 0.056) and timing of last intake (ρ = 0.36, *p* = 0.062).

In adjusted multivariable regression models, we examined the associations between dietary intake behaviors and salivary outcomes, including xerostomia severity and salivary flow measures. All models were adjusted for age, biological sex, BMI, total number of medications, ADS, diabetes, thyroid or endocrine disorders, and gastrointestinal conditions. XI was significantly associated with dietary timing behaviors and ADS in adjusted models (Table 3). The later timing of last intake (β = 0.63, *p* = 0.032) and later timing of last snack (β = 0.65, *p* = 0.004) were independently associated with higher XI scores, and higher anticholinergic burden was also independently associated with greater xerostomia severity (β ≈ 0.92, *p* ≈ 0.01).

No dietary intake variables were significantly associated with UWS or MSF rates in adjusted linear regression models (Supplementary Table S1). In adjusted multivariable logistic regression analyses examining UWS status (normosalivation vs. hyposalivation), later timing of first intake was associated with lower odds of normosalivation (OR = 0.62, 95% CI 0.40–0.95, *p* = 0.028) (Table 4). Male sex (OR ≈ 14.0, *p* < 0.01) and the presence of gastrointestinal disease (OR ≈ 15.3, *p* = 0.005) were also significant predictors of UWS status. Adjusted models demonstrated good discrimination, with area under the receiver operating characteristic curve (AUC) values ranging from approximately 0.87 to 0.88.

## 4. Discussion

This exploratory study aimed to investigate whether habitual water and beverage consumption, and dietary timing, were associated with xerostomia severity and salivary gland function among individuals with medication-induced dry mouth. Our findings partially support an association between intake timing and xerostomia severity: the later timing of the last intake and last snack was associated with more severe xerostomia symptoms. These findings align with prior observations that individuals reporting dry mouth may have irregular or delayed eating patterns [16]. Such patterns are consistent with the characterization of pharmacological thirst, in which the medication-induced blockade of cholinergic signaling leads to oral dryness that can alter eating behavior. Within this conceptual framework, xerostomia may influence the structure of daily intake independent of hydration needs, potentially contributing to delayed or fragmented eating routines. Moreover, Lee et al. [22] and Shiota et al. [36] highlighted that oral dryness in older adults is closely associated with systemic health conditions and reduced oral health-related quality of life, factors that may further disrupt intake timing and daily routines.

We found that participants with more severe salivary dysfunction, whether measured objectively by unstimulated whole or minor salivary gland secretion, or subjectively via the Xerostomia Inventory, did not consume significantly more water or total beverages. The absence of increased fluid intake contrasts with the common assumption that oral dryness prompts compensatory hydration behavior. Experimental studies further show that reduced oral moisture can alter drinking patterns, such as increasing the frequency or duration of sips, without increasing total volume consumed [37]. When salivary output is low, drinking may offer only transient relief, leading to repeated small-volume intake episodes rather than sustained fluid replacement. This may help explain why greater xerostomia severity in our cohort did not correspond to greater beverage consumption. Indeed, Lee et al. [22] found that older adults with dry mouth reported lower overall water intake in free-living populations.

Participants who reported eating at night had a significantly higher anticholinergic burden. Because salivary secretion is already reduced during sleep [38], individuals taking xerogenic medications may be particularly prone to oral dryness during the night. Jacob et al. [39] reported that nighttime dry mouth complaints were significantly associated with the number of xerogenic medications taken and specifically noted that ”xerogenic drugs taken at night may be more likely to cause dry mouth, due to reduced salivary flow during sleep”. Although formal dosing guidelines were not provided, the authors emphasized the importance of considering symptom timing in medication management. In this context, nighttime meal consumption may reflect a compensatory response to nocturnal discomfort among individuals exposed to xerogenic medications.

An earlier timing of the first intake was associated with higher odds of normosalivation, suggesting that the timing of morning meals may relate to underlying salivary function. Circadian studies have shown that salivary flow is lowest during the night and early morning hours, gradually increasing throughout the day [40], which may contribute to morning oral dryness. In population-based research, individuals with xerostomia frequently report impaired oral function upon waking, including difficulties with speaking, eating, or swallowing [41]. Taken together, these findings support the idea that delayed morning intake may reflect greater discomfort or functional limitation associated with low morning salivary output.

In individuals with severe hyposalivation (unstimulated salivary flow ≤ 0.1 mL/min), modest associations emerged between salivary output and dietary behavior that were not observed in the rest of the sample. Specifically, lower UWS was correlated with a later timing of the last meal, and xerostomia severity showed trends toward higher beverage volume and delayed intake. These patterns may reflect behavioral adaptation to persistent oral dryness, particularly late at night, when salivary flow is severely diminished [21,40]. The absence of such associations among participants with higher salivary flow suggests that symptom severity plays a key role in shaping how and when individuals eat and drink. While the sensation of oral dryness typically decreases during eating, Villa et al. [42] noted that individuals with dry mouth often feel the urge to drink frequently while eating to facilitate swallowing, which may influence the timing of intake. Our exploratory findings point to a symptom-driven behavioral pattern of xerostomia in individuals with marked salivary hypofunction, suggesting that salivary flow thresholds may be considered for dietary assessment and support.

This study has several limitations. As a cross-sectional, exploratory analysis, the findings cannot establish causal relationships between intake timing, salivary function, and xerostomia severity. The absence of a control group limits generalizability and prevents determining whether the observed behavioral patterns are specific to xerostomia. Additional limitations include a modest sample size, the lack of objective validation of self-reported behaviors, and the potential for recall or reporting bias. Future studies incorporating objective measures of intake would enhance the methodological robustness. In the present study, beverage intake was analyzed primarily by total volume, without stratification by beverage type. Different beverage types, including caffeinated, sugar-sweetened, and alcoholic beverages, may differentially influence hydration status and oral dryness, and these effects may also interact with anticholinergic medications. We did not assess the time required to consume meals, which may be prolonged in individuals who modify food textures, chew slowly, or eat cautiously to avoid aspiration. Intake timing was self-reported, and swallowing function was not evaluated. Several factors known to influence salivary flow, such as hydration status, nutritional condition, and the sensory characteristics of food and beverages, were not measured. As objective hydration markers were not assessed in this study, the interpretation of the relationship between fluid intake, physiological hydration, and xerostomia severity cannot be fully elucidated. Because the study focused on individuals with medication-induced xerostomia, the lack of a non-xerostomic control group limits inference beyond this population. In addition, the timing of medication administration was not recorded, restricting the ability to evaluate possible interactions between drug effects and the diurnal pattern of symptoms.

## 5. Conclusions

This study demonstrates that, among individuals with medication-induced xerostomia, the timing of intake, rather than total fluid volume, is meaningfully associated with perceived dry mouth severity and anticholinergic medication burden. These findings suggest adaptive responses to oral dryness, particularly in the evening and at night. At the same time, total water and beverage intake did not correspond to salivary status, indicating that hypofunction does not necessarily reflect changes in water or beverage intake. By incorporating both objective salivary measures and validated symptom assessments, our exploratory study highlights the relevance of intake timing as a behavioral marker in xerostomia research. Future studies, including appropriate control groups and objective hydration status, should examine whether targeted adjustments to dietary timing can support symptom management and improve quality of life in this population.

## Figures and Tables

**Table 1:** Participant characteristics by hyposalivation status (*n* = 90).

	Hyposalivation (≤0.3 mL/min) *n* = 63	Normosalivation (>0.3 mL/min) *n* = 27		
Variable	Value = *n* (%) or Median [IQR]	Value = *n* (%) or Median [IQR]	Statistical Test	*p*-Value
Age (years)	59.00 [7.50]	57.00 [9.00]	Mann–Whitney U	0.0867
Female	45 (71.4%)	17 (63.0%)	Chi-square	0.440
BMI	30.66 [8.73]	28.97 [8.00]	Mann–Whitney U	0.1033
ADS	3.00 [4.00]	3.00 [3.00]	Mann–Whitney U	0.2728
Total number of medications	12.00 [9.00]	8.00 [9.50]	Mann–Whitney U	**0.024**
Diabetes	17 (27.0%)	3 (11.1%)	Fisher’s Exact	0.1648
Cardiac diseases	11 (17.5%)	3 (11.1%)	Fisher’s Exact	0.5405
Circulatory diseases	28 (44.4%)	13 (48.1%)	Fisher’s Exact	0.8192
Endocrine/hormonal diseases	15 (23.8%)	1 (3.7%)	Fisher’s Exact	**0.0323**
Gastrointestinal diseases	19 (30.2%)	9 (33.3%)	Fisher’s Exact	0.8066
UWS	0.11 [0.13]	0.59 [0.25]	Mann–Whitney U	**<0.001**
MSF	3.50 [3.00]	6.50 [4.25]	Mann–Whitney U	**<0.001**
XI	37.00 [11.50]	31.00 [10.5]	Mann–Whitney U	**0.0326**

Abbreviations: ADS = Anticholinergic Drug Scale; BMI = body mass index; MSF = Minor Salivary Gland Flow Rate (mL/cm^2^/min); UWS = unstimulated whole saliva flow rate (mL/min); XI = Xerostomia Inventory. Hyposalivation was defined by the unstimulated whole saliva flow rate. Bolded *p*-values indicate statistically significant differences (*p* < 0.05).

**Table 2:** Dietary timing and beverage intake characteristics by salivary flow status.

	Hyposalivation (≤0.3 mL/min)	Normosalivation (>0.3 mL/min)		
Variable	Value = *n* (%) or Median [IQR]	Value = *n* (%) or Median [IQR]	Statistical Test	*p*-Value
Daily Water Intake (mL)	48.00 [48.00]	48.00 [87.00]	Mann–Whitney U	0.811
Total Beverage Volume (mL)	86.29 [72.57]	85.14 [170.58]	Mann–Whitney U	0.7513
Total kcal from Beverages	123.33 [315.08]	127.93 [426.61]	Mann–Whitney U	0.5911
First Intake (h)	9.0 [8.0–10.0]	8.5 [6.75–9.5]	Mann–Whitney U	0.2657
Last Intake (h)	20.0 [19.0–21.0]	20.0 [18.75–21.0]	Mann–Whitney U	0.8237
Snack Count	2.0 [1.0–2.0]	2.0 [0.5–3.0]	Mann–Whitney U	0.8944
Last Snack Time (h)	20.00 [2.00]	20.00 [1.50]	Mann–Whitney U	0.3457
Intake After 9 p.m.	14 (22.2%)	7 (25.9%)	Fisher’s Exact	0.7873
Nighttime Intake (Waking up to Eat)	8 (12.7%)	4 (14.8%)	Fisher’s Exact	0.7472
Intake Range (h)	11.50 [2.50]	11.00 [3.25]	Mann–Whitney U	0.6141
Intake Window Type = Long	4 (6.3%)	3 (11.1%)	–	–
Intake Window Type = Moderate	26 (41.3%)	9 (33.3%)	–	–
Intake Window Type = Short	33 (52.4%)	15 (55.6%)	–	–
Intake Window Type: Test	–	–	Chi-square	0.6399
Total Intake Events	4.00 [1.00]	4.00 [3.00]	Mann–Whitney U	0.9567
Irregular Food Timing (>2 h Shift)	2 (3.2%)	3 (11.5%)	Fisher’s Exact	0.1466

Abbreviations: IQR, interquartile range; mL, milliliters; kcal, kilocalories; h, hour. Hyposalivation was defined by the unstimulated whole saliva flow rate.

**Table 3:** Multivariable linear regression models examining associations between dietary intake behaviors and xerostomia severity.

Outcome: Xerostomia Severity (XI)					
Predictor	Beta (β)	SE	95% CI Lower	95% CI Upper	*p*-Value
*Dietary Intake Variables*					
First Intake (h)	0.15	0.55	−0.95	1.25	0.784
Last Intake (h)	0.63	0.29	0.05	1.21	**0.032**
Last Snack Time (h)	0.65	0.22	0.21	1.09	**0.004**
Intake Range (h)	0.13	1.09	−2.01	2.27	0.908
Intake Window Type (Moderate vs. Long)	6.1	6.25	−6.26	18.46	0.332
Intake Window Type (Short vs. Long)	6.93	8.13	−9.15	23.01	0.397
Total Intake Events	−3.2	2.78	−8.68	2.28	0.255
Irregular Food Timing (>2 h Shift)	−5.91	5.77	−17.24	5.43	0.31
Daily Water Intake (mL)	0.0004	0.0034	−0.006	0.007	0.904
Total Beverage Volume (mL)	−0.00009	0.0018	−0.004	0.004	0.963
Total kcal from Beverages	0.0038	0.0032	−0.002	0.01	0.228
*Key Covariates*					
ADS	0.92	0.35	0.23	1.61	**0.01**
Age (Years)	−0.37	0.19	−0.75	0.02	0.06
Gastrointestinal Disease (Yes vs. No)	2.74	0.98	0.81	4.66	0.005

Values are presented as regression coefficients (β) with standard errors (SE), 95% confidence intervals (CI), and *p*-values; bolded *p*-values indicate statistical significance at *p* < 0.05. Abbreviations: XI, Xerostomia Inventory; β, regression coefficient; SE, standard error; CI, confidence interval; ADS, Anticholinergic Drug Scale.

**Table 4:** Multivariable logistic regression model examining predictors of UWS status.

Outcome: UWS Status (Normosalivation vs. Hyposalivation)				
Predictor	Odds Ratio (OR)	95% CI Lower	95% CI Upper	*p*-Value
First intake (h)	0.62	0.4	0.95	**0.028**
Age (years)	0.84	0.72	0.97	0.022
BMI	0.93	0.81	1.07	0.319
ADS	0.8	0.57	1.1	0.168
Total number of medications	0.97	0.85	1.11	0.673
Male sex (vs. female)	13.96	2.49	78.28	**0.003**
Diabetes (yes vs. no)	0.3	0.04	2.21	0.236
Endocrine/thyroid disease (yes vs. no)	0.08	0.01	1.1	0.059
Gastrointestinal disease (yes vs. no)	15.41	2.25	105.71	**0.005**
**Model performance: area under the ROC curve (AUC) = 0.884**				

Values are presented as odds ratios (OR) with 95% confidence intervals (CI) and *p*-values; bolded *p*-values indicate statistical significance at *p* < 0.05. Hyposalivation was defined as unstimulated whole saliva (UWS) flow ≤ 0.3 mL/min, and normosalivation was defined as UWS flow > 0.3 mL/min. Model performance is summarized using the area under the receiver operating characteristic curve (AUC). Abbreviations: OR, odds ratio; CI, confidence interval; ADS, Anticholinergic Drug Scale; BMI, body mass index.

## Data Availability

The datasets used and/or analyzed during the current study are available from the corresponding author on reasonable request.

## Supplementary Materials

The following supporting information can be downloaded at: https://www.mdpi.com/article/10.3390/nu18040661/s1, Table S1: Multivariable Linear Regression Models Examining Associations Between Dietary Intake Behaviors and Salivary Flow Rates.

## Abbreviations

The following abbreviations are used in this manuscript:

ADS	Anticholinergic Drug Scale
BEVQ	Beverage Intake Questionnaire
BMI	Body Mass Index
EIOH	Eastman Institute for Oral Health
FTS	Food Timing Screener
IRB	Institutional Review Board
MSF	Minor Salivary Flow
UWS	Unstimulated Whole Saliva
URMC	University of Rochester Medical Center
XI	Xerostomia Inventory
