# Supplementary material for: Exploring Beverage Intake and Dietary Timing Patterns in Medication-Induced Xerostomia: A Cross-Sectional Pilot Study
Source: Nutrients. 2026 Feb 18;18(4):661. doi: 10.3390/nu18040661 (PMC12943099; doi:10.3390/nu18040661)
Supplement: Supplementary file 1 [file nutrients-18-00661-s001.zip › Supplementary Table S1.pdf]

Supplementary Table S1. Multivariable Linear Regression Models Examining Associations Between Dietary Intake Behaviors and Salivary Flow Rates

Outcome: Unstimulated whole saliva (UWS, ml/min)

Predictor	Beta (β)	SE	95% CI Lower	95% CI Upper	p-value
First intake time (hrs)	-0.02605	0.0298	-0.084458	0.032358	0.385
Last intake time (hrs)	0.01856	0.0162	-0.013192	0.050312	0.255
Snack count	0.01746	0.0931	-0.165016	0.199936	0.852
Last snack time (hrs)	0.00155	0.0138	-0.025498	0.028598	0.911
Intake range (hrs)	0.02435	0.0316	-0.037586	0.086286	0.444
Intake window (moderate vs	-0.24074	0.1819	-0.597264	0.115784	0.19
Intake window (short vs long	0.01672	0.2367	-0.447212	0.480652	0.944
Total intake events	0.01752	0.081	-0.14124	0.17628	0.829
Irregular food timing (>2h)	0.16765	0.1679	-0.161434	0.496734	0.322
Water intake (ml)	-4.44E-05	0.000098	-0.00023648	0.00014768	0.652
Total beverage volume (ml)	0.0000364	0.000053	-0.00006748	0.00014028	0.494
Total kcal from beverages	0.0000323	0.0000918	-0.000147628	0.00021223	0.726

Outcome: Minor salivary gland flow (MSF, µl/cm²/min)

Predictor	Beta (β)	SE	95% CI Lower	95% CI Upper	p-value
First intake time (hrs)	-0.20177	0.32105	-0.831028	0.427488	0.532
Last intake time (hrs)	0.0893	0.17422	-0.2521712	0.4307712	0.61
Snack count	-0.17811	1.00314	-2.1442644	1.7880444	0.86
Last snack time (hrs)	0.01182	0.14825	-0.27875	0.30239	0.937
Intake range (hrs)	-0.08421	0.34095	-0.752472	0.584052	0.806
Intake window (moderate vs	-0.92396	1.95996	-4.7654816	2.9175616	0.639
Intake window (short vs long	0.06361	2.55078	-4.9359188	5.0631388	0.98
Total intake events	-0.06635	0.87291	-1.7772536	1.6445536	0.94
Irregular food timing (>2h)	2.42698	1.80954	-1.1197184	5.9736784	0.185
Water intake (ml)	-0.000421	0.00106	-0.0024986	0.0016566	0.691
Total beverage volume (ml)	0.000247	0.000571	-0.00087216	0.00136616	0.667
Total kcal from beverages	0.00149	0.000989	-0.00044844	0.00342844	0.136
